# Supplementary material for: Combined KRAS-MAPK pathway inhibitors and HER2-directed drug conjugate is efficacious in pancreatic cancer
Source: Nat Commun. 2024 Mar 20;15:2503. doi: 10.1038/s41467-024-46811-w (PMC10954758; doi:10.1038/s41467-024-46811-w)
Supplement: Supplementary file 1 — Supplementary Information [file 41467_2024_46811_MOESM1_ESM.docx]

**Combined KRAS-MAPK pathway inhibitors and HER2-directed drug conjugate is efficacious in pancreatic cancer**

Ashenafi Bulle^1,#^, Peng Liu^1,2,#^, Kuljeet Seehra^1^, Sapana Bansod^1^, Yali Chen^1^, Kiran Zahra^1^, Vikas Somani^1^, Iftikhar Ali Khawar^1^, Hung-Po Chen^1^, Paarth B. Dodhiawala^1^, Lin Li^1^, Yutong Geng^1^, Chia-Kuei Mo^1^, Jay Mahsl^1^, Li Ding^1^, Ramaswamy Govindan^1^, Sherri Davies^1^, Jacqueline Mudd^3^, William G. Hawkins^3^, Ryan C. Fields^3^, David G. DeNardo^1^, Deborah Knoerzer^4^, Jason M. Held^1^, Patrick M. Grierson^1^, Andrea Wang-Gillam^1^, Marianna B. Ruzinova^5^, Kian-Huat Lim^1,^*. (^#^equal contribution)

^1^Division of Oncology, Department of Internal Medicine, Washington University School of Medicine, St. Louis, MO 63110, USA

^2^Department of General Surgery, Shengjing Hospital of China Medical University, Shenyang, China

^3^Section of Hepatobiliary Surgery, Department of Surgery, Washington University School of Medicine, St. Louis, MO 63110, USA

^4^BioMed Valley Discoveries, Kansas City, MO 64111, USA

^5^Department of Pathology and Immunology; Washington University School of Medicine, St. Louis, MO 63110, USA

**SUPPLEMENTARY INFORMATION**

**This file includes 9 Supplementary Figures, 9 Supplementary Figure Legends**

**and 4 Supplementary Tables**

***Corresponding author:**

Kian-Huat Lim, MD, PhD

Washington University School of Medicine

660 South Euclid Avenue

Campus Box 8069

Saint Louis, MO 63110

Tel: 314-362-6157

Fax: 314-747-9329, Email: [kian-huat.lim@wustl.edu](mailto:kian-huat.lim@wustl.edu)

**Supplementary Figures and Legends**

**Supplementary Figure 1. Targeting the MAPK pathway is compromised by HER2 activation.**

**a,** Western blots showing dose-dependent increase of phosphorylated ERK1/2, p90RSK, and cleaved caspase-3 levels in PDAC cells treated with the indicated concentrations of gemcitabine for 24 h. **b,** Densitometric quantification of serial p-ERK1/2:GAPDH and p-p90RSK:total p90RSK ratios of the indicated cells lines treated with different concentrations of gemcitabine for 24 h. **c,** Representative IHC images and quantification using ImageJ software show increased p-ERK1/2 staining in different xenograft tumours treated with vehicle or gemcitabine 100 mg/kg by intraperitoneal injection for ~2 weeks. All tumours were harvested ~4 h after the last injection. Scale bar =100 μm. N= 30 fields taken (5 tumours with 6 fields per tumour). Data are presented as mean±SEM. *P*-values were calculated using two-tailed unpaired t-test with Welch’s correction. ****, *p* < 0.0001. Whiskers show min/max value, the box boundaries show the first and third quartiles and the central lines correspond to the median. **d,** Western blots showing dose-dependent changes of phosphorylated ERK1/2, p90RSK, and cleaved caspase-3 levels in different PDAC cells treated with the indicated concentrations of ulixertinib for 24 h. Both **a** and **d** were conducted two times, and one set of data was presented. **e,** Densitometric quantification of serial p-ERK1/2: GAPDH and p-p90RSK: p90RSK ratios of the indicated cells lines treated with different concentrations of ulixertinib for 24 h. **f,** Median effect analyses of ulixertinib or trametinib in combination with gemcitabine in five indicated PDAC lines as represented by combination indices (CI) calculated using Compusyn software. Cells were cultured in triplicates in six fixed-ratio concentrations (all in μM) of ulixertinib: gemcitabine (10:10, 5:5, 2.5:2.5, 1.25:1.25; 0.625:0.625, 0.31:0.31) for 3 days and viability measured by Alamar Blue assay. Four independent experiment was performed. **g,** Flow cytometric plots and quantification of apoptotic Pa01c cells defined by positive Annexin-V staining following treatment as indicated for 24 h. Data presented as mean ± SEM of three independent experiments, one-way ANOVA followed by Tukey’s multiple comparison test (1µM ulixertinib + 2.5µM gemcitabine vs vehicle, *p*<0.0001) and (1µM Ulixertinib + 2.5µM Gemcitabine vs 1µM Ulixertinib, *p*=0.0001). **h,** Western blots showing different levels of p-HER2 and p-ERK1/2 in different PDAC cells cultured in the low serum (0.5% FBS). This experiment was conducted two times, and one set of data was presented. **i,** Representative IHC images showing changes in protein expression of expression of total HER2, p-ERK1/2, DUSP4 and DUSP6 in the indicated xenograft tumours harvested from mice treated with vehicle (PBS) or ulixertinib (100mg/kg/day oral gavage) for 2 weeks. Scale bar=100µm. Tumours were harvested 4 h after the last dose of ulixertinib. Source data are provided as a Source Data file.

**Supplementary Figure 1**

**
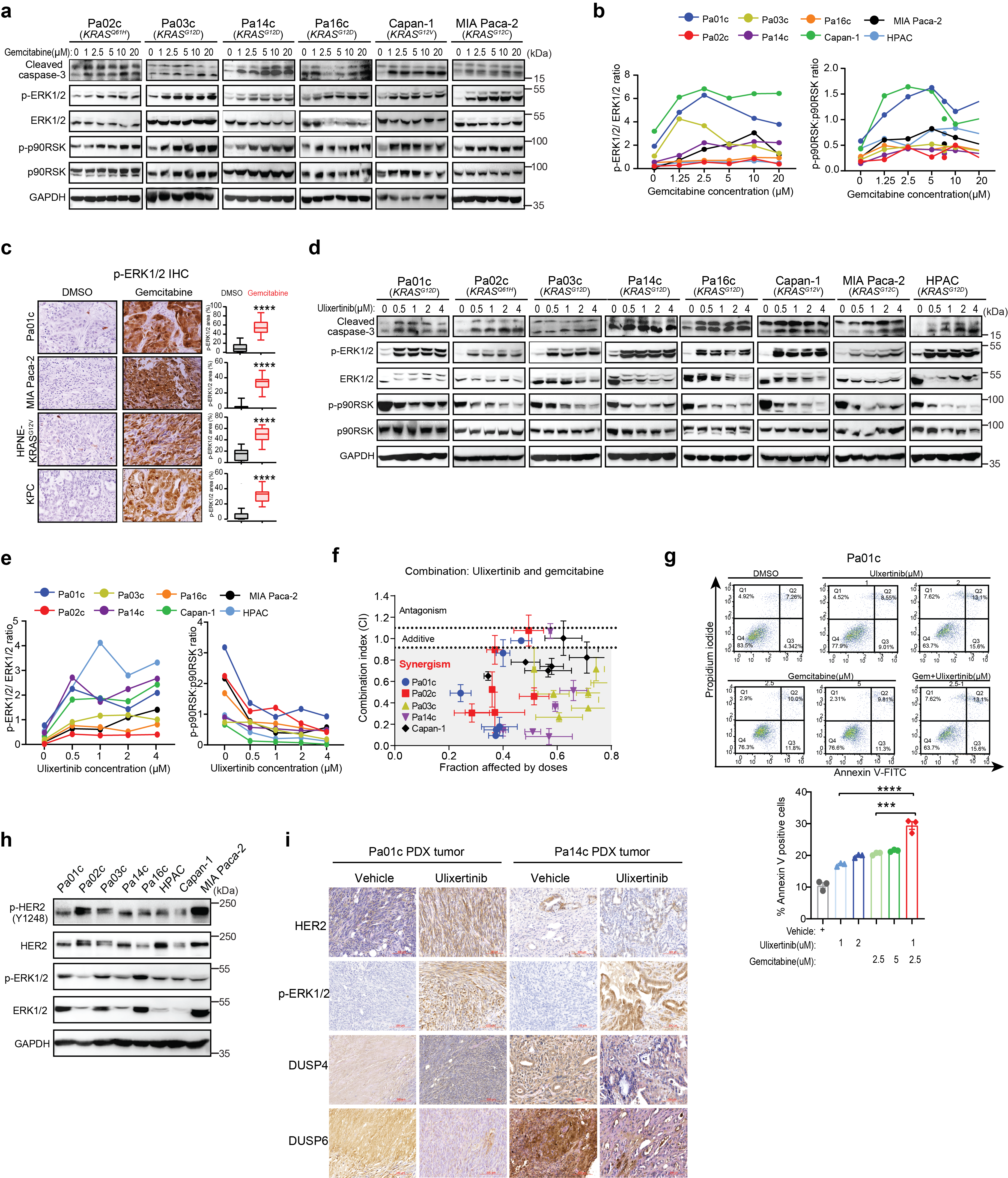
**

**Supplementary Figure 2. Targeting the MAPK pathway is inadequate and compromised by HER2 activation.**

**a,** Heat map illustrating fold change in apoptosis relative to that of ulixertinib or trametinib alone, as measured by flow cytometric Annexin-V staining of different PDAC cells after 24-hour treatment with the indicated drug combinations. The drug concentrations used were as follows: ulixertinib (1μM), trametinib (0.5μM), afatinib (0.5μM), MMP-inhibitor (Ro 28–2653 0.1μM), MUC1 inhibitor (GO201, 2μM), and JAK inhibitor (Ruxolitinib, 1μM). **b,** Quantification of apoptotic Pa01c and HPAC cells defined by positive Annexin-V staining following treatment as indicated for 24 h. Data from **a** and **b** were generated from three independent experiments. Data presented as mean ± SEM, one-way ANOVA followed by Tukey’s multiple comparison test. For Pa01c: ulixertinib vs vehicle (*p*=0.9027), afatinib vs vehicle (*p*=0.4880), afatinib + ulixertinib vs ulixertinib (*p*<0.0001), afatinib + ulixertinib vs afatinib (*p*<0.0001), MMPi vs vehicle (*p*<0.0001), MMPi + ulixertinib vs ulixertinib (*p*<0.0001), MMPi + ulixertinib vs MMPi (*p*=0.9999), MUC1i vs vehicle (*p*<0.0001), MUC1i + ulixertinib vs ulixertinib (*p*<0.0001), MUC1i + ulixertinib vs MUC1i (*p*=0.0020), ruxolitinib vs vehicle (*p<*0.0001), ruxolitinib + ulixertinib vs ulixertinib (*p*<0.0001), ruxolitinib + ulixertinib vs ruxolitinib (*p*=0.0006); trametinib vs vehicle (*p*=0.3826), afatinib vs vehicle (*p*=0.4525), afatinib +trametinib vs trametinib (*p*<0.0001), afatinib + trametinib vs afatinib (*p*<0.0001), MMPi vs vehicle (*p*<0.0001), MMPi + trametinib vs trametinib (*p*<0.0001), MMPi +trametinib vs MMPi (*p*=0.9560), MUC1i vs vehicle (*p*=0.0541), MUC1i +trametinib vs trametinib (*p*=0.0188), MUC1i + trametinib vs MUC1i (*p*=0.1847), ruxolitinib vs vehicle (*p*<0.0001), ruxolitinib + trametinib vs trametinib (*p*<0.0001), ruxolitinib +trametinib vs ruxolitinib (*p*=0.1934) and HPAC: ulixertinib vs vehicle (*p*=0.9937), afatinib vs vehicle (*p*=0.9331), afatinib + ulixertinib vs ulixertinib (*p*<0.0001), afatinib + ulixertinib vs afatinib (*p*<0.0001), MMPi vs vehicle (*p*=0.9983), MMPi + ulixertinib vs ulixertinib (*p*<0.0001), MMPi + ulixertinib vs MMPi (*p*<0.0001), MUC1i vs vehicle (*p*=0.9999), MUC1i + ulixertinib vs ulixertinib (*p*<0.0001), MUC1i + ulixertinib vs MUC1i (*p*<0.0001), ruxolitinib vs vehicle (*p*=0.6381), ruxolitinib +ulixertinib vs ulixertinib (*p*=0.4576), ruxolitinib + ulixertinib vs ruxolitinib (*p*=0.9643); trametinib vs vehicle (*p*=0.4361), afatinib vs vehicle (*p*=0.8843), afatinib + trametinib vs trametinib (*p*<0.0001), afatinib + trametinib vs afatinib (*p*<0.0001), MMPi vs vehicle (*p*=0.9964), MMPi + trametinib vs trametinib (*p*<0.0001), MMPi + trametinib vs MMPi (*p*<0.0001), MUC1i vs vehicle (*p*=0.9999), MUC1i +trametinib vs trametinib (*p*=0.2530), MUC1i + trametinib vs MUC1i (*p*=0.0009), ruxolitinib vs vehicle (*p*=0.5032), ruxolitinib + trametinib vs trametinib (*p*=0.9999), ruxolitinib + trametinib vs ruxolitinib (*p*=0.7523). **c,** Median effect analyses of Ulixertinib or Trametinib in combination with different indicated agents in seven PDAC lines as represented by combination indices (CI) calculated using Compusyn software. Cells were cultured in triplicate at six fixed-ratio concentrations for three days, and viability was measured using the Alamar Blue assay. The concentrations of each agent are provided in the **Supplementary Table 4**. (**a**, **b**) are representative of five independent experiments and (**c**) is representative of three independent experiments. *p* values are calculated using two-way ANOVA (**b**) and one-way ANOVA (**a**) with Dunnet’s post-test. Ns: not significant, *, *p* < 0.05, **, *p* < 0.01, ***, *p* < 0.001 and ****, *p*<0.0001. **d,** Dose-dependent curves for the inhibitory effect of ulixertinib or trametinib in the indicated scramble or *ERBB*-silenced Pa01c or HPAC cell lines after 5 days of treatment. Three independent experiments were performed, yielding similar results, and IC_50_ values were obtained from one set. **e,** Kaplan-Meier survival analysis of TCGA database showing overall survival (OS) and relapse-free survival (RFS) of PDAC patients after pancreatic cancer resection stratified by high vs. low median *ERBB2* mRNA expression. **f,** Western blots showing changes of different DUSP family members in 293T cells following 16 h treatment with two different concentrations of ulixertinib. **g,** Western blots showing time-dependent changes of DUSP4 and DUSP6 levels following treatment with two different concentrations of ulixertinib for different durations in Pa01c cells. The bar graph includes quantitative changes of DUSP4 or DUSP6 protein levels from three independent experiments. Data presented as mean ± SEM, one-way ANOVA followed by Tukey’s multiple comparison test; 1µM and 2µM ulixertinib concentrations do not reduce DUSP4 and 6 significantly within the first three hrs of treatment, except 2µM ulixertinib on DUSP4 after 3hrs (*p*=0.0499). DUSP4 ( after 6hrs of treatment with 1µM ulixertinib (*p*=0.0037) and 2µm ulixertinib (*p*=0.0042), after 12hrs of treatment with 1µm ulixertinib (*p*=0.0016) and 2µm ulixertinib (*p*=0.0009), after 24hrs of treatment with 1µm ulixertinib (*p*=0.0016) and 2µm ulixertinib (*p*=0.0004); dusp6 (after 6hrs of treatment with 1µm ulixertinib (*p*=0.4786) and 2µm ulixertinib (*p*=0.6851), after 12hrs of treatment with 1µm ulixertinib (*p*=0.0152) and 2µm ulixertinib (*p*=0.0156), after 24hrs of treatment with 1µm ulixertinib (*p*=0.0091) and 2µm ulixertinib (*p*=0.0050). **f** was conducted two times, and **g** conducted three times, in both one set of data was presented. Source data are provided in Source Data file.

**Supplementary Figure 2**

**
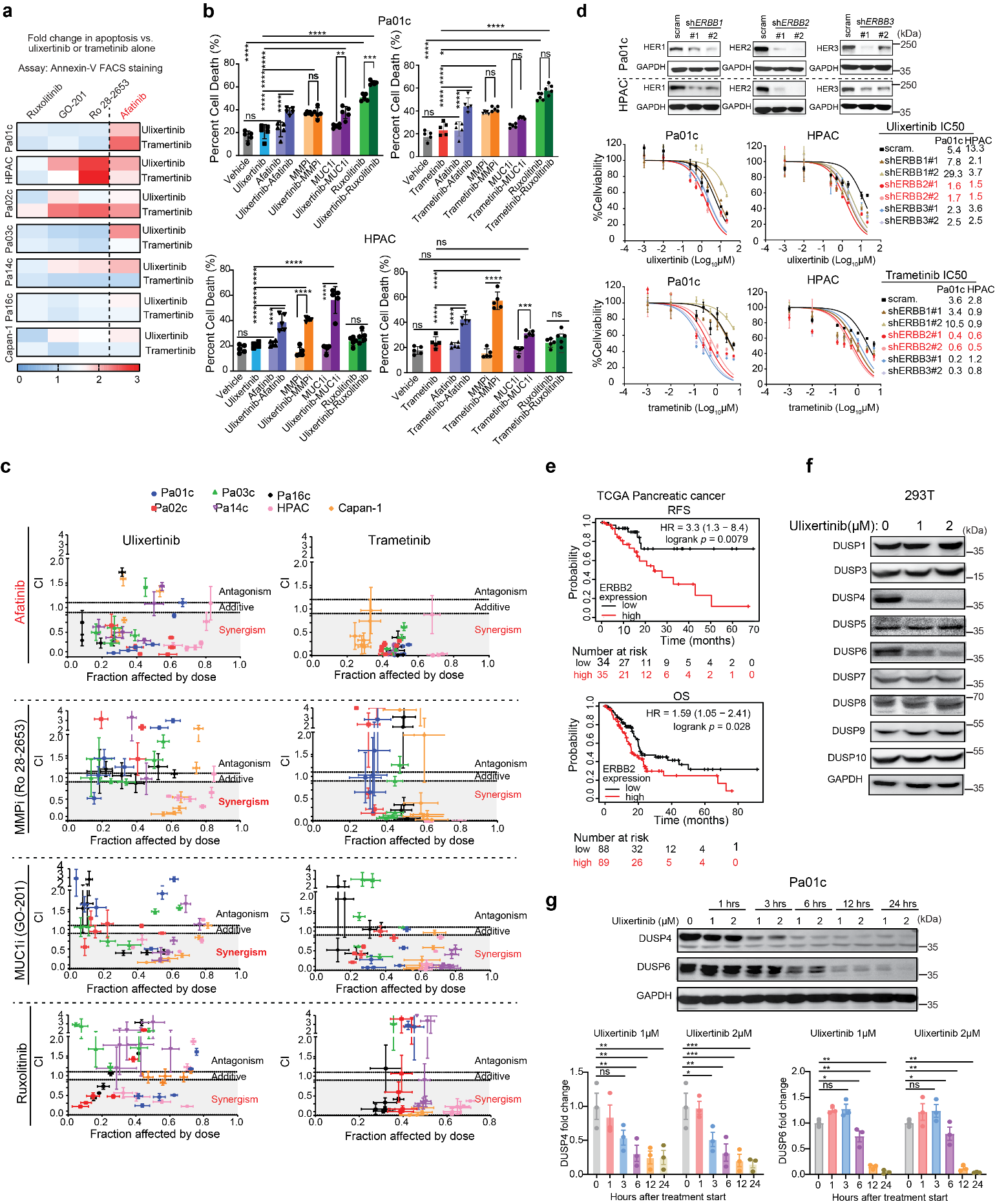
**

**Supplementary Figure 3. HER2 drives DUSP6 degradation.**

**a,** Western blots showing changes in DUSP4 and DUSP6 levels in 293T cells co-transfected with HER1/HER2, HER1/HER3, or HER2/HER3 for 36 h and then treated with DMSO or afatinib at 1μM for 16 h. **b,** Immunoprecipitation (IP) experiment in 293T cells showing polyubiquitination of ectopically expressed HA-tagged DUSP6 in the presence or absence of co-transfected FLAG-tagged HER2 followed by co-treatment with DMSO or bortezomib for 6 h. WCL: Whole cell lysates. Both **a** and **b** were conducted two times, and one set of data was presented. Source data are provided in Source Data file.

**Supplementary Figure 3**

**
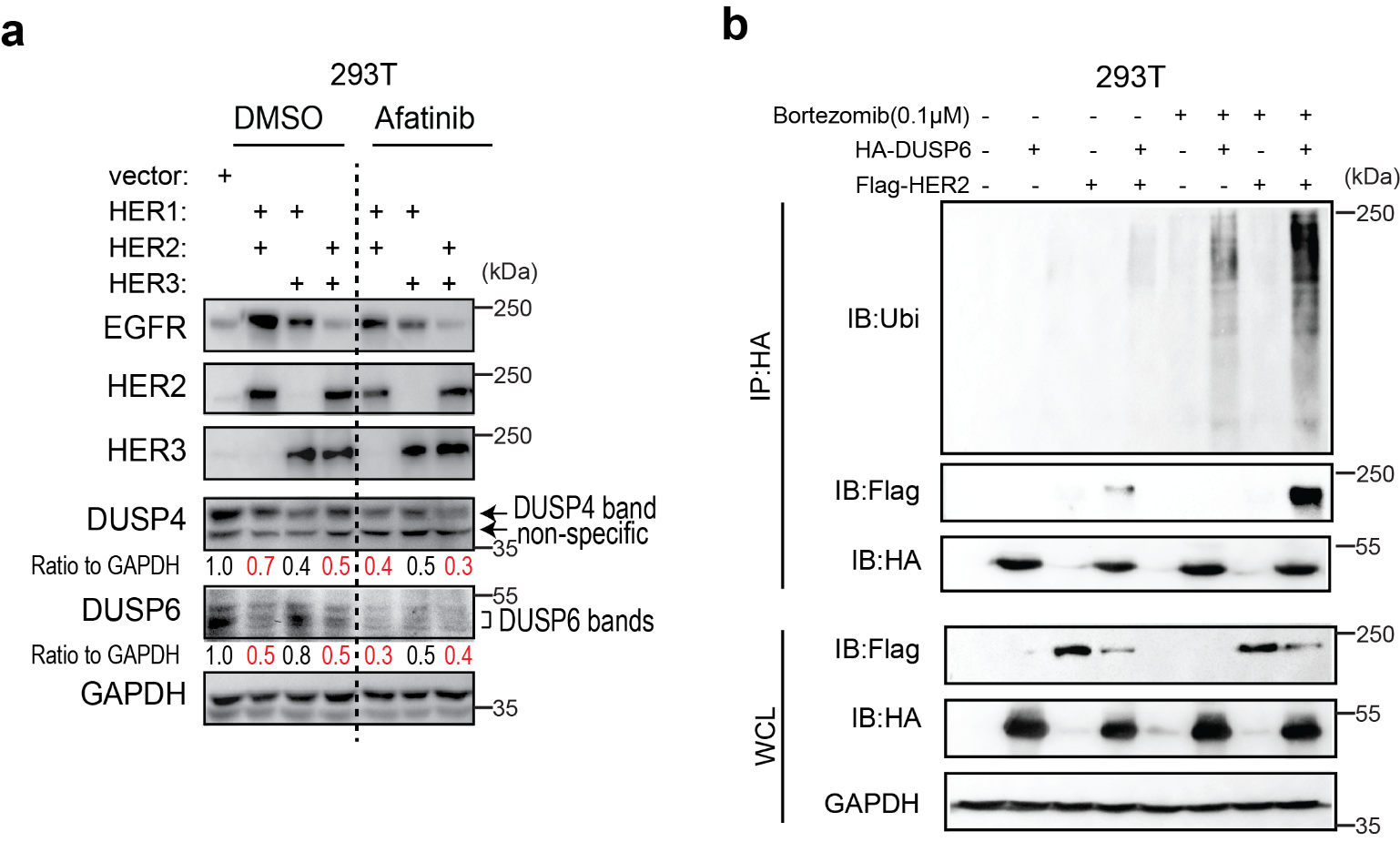
**

**Supplementary Figure 4. E3 ligaseTRIM21 regulates DUSP6 stability.**

**a,** Western blots and quantification showing time-dependent downregulation of DUSP6 protein level in DMSO or MRTX1133-pretreated HPAC cells after exposure to ulixertinib 2μM for the indicated durations. **b,** IP experiment in 293T cells showing polyubiquitination of ectopically expressed FLAG-tagged DUSP6 in the absence or presence of co-transfected GFP-tagged TRIM21, treated for 16 h with trametinib or ulixertinib at the indicated concentrations. Both **a** and **b** were conducted two times, and one set of data was presented. Source data are provided as a Source Data file.

**Supplementary Figure 4**

**
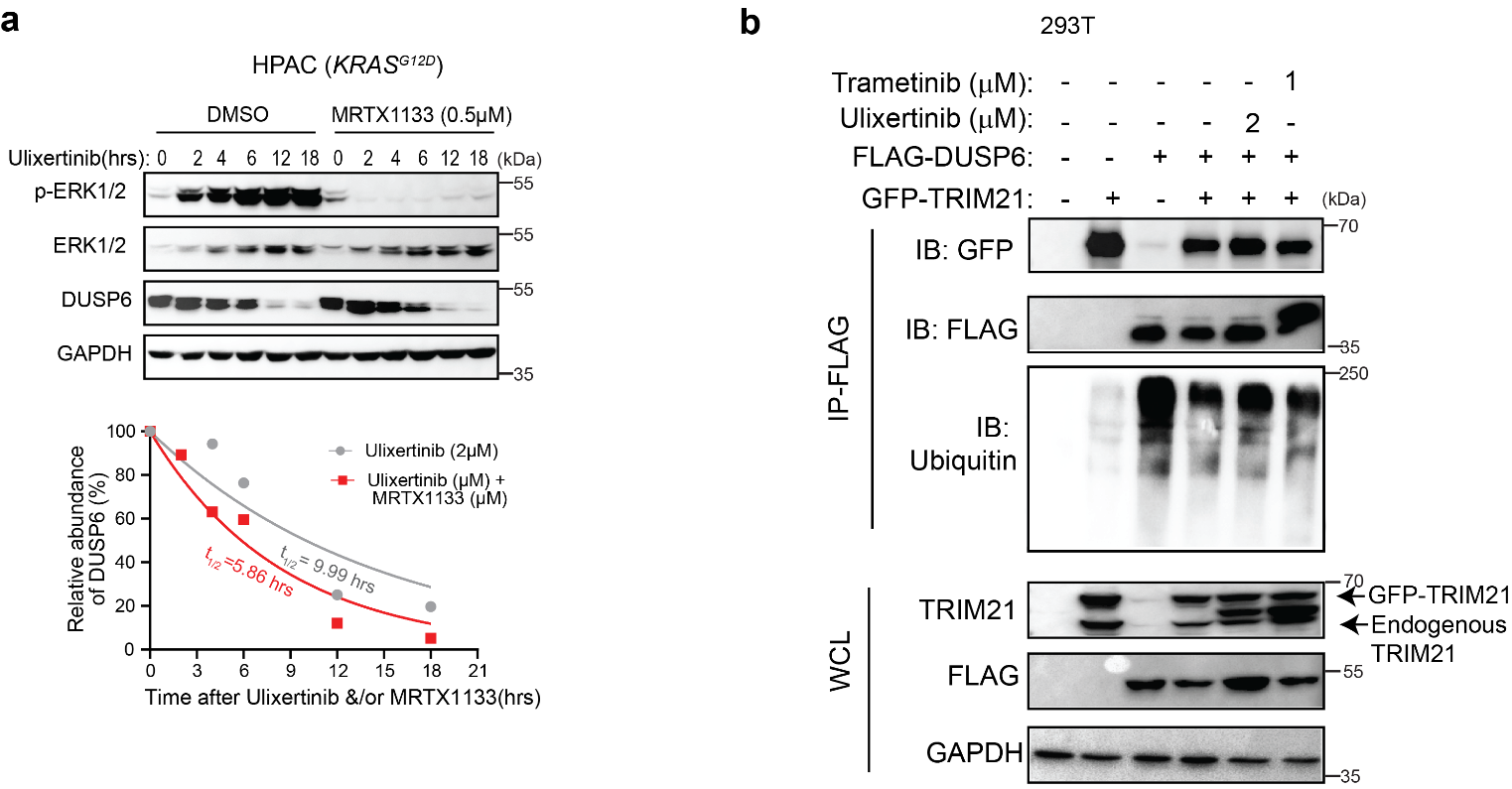
**

**Supplementary Figure 5. Targeted MAPK-based combinations require chemotherapy to achieve meaningful therapeutic efficacy.**

**a,** Growth kinetics of 16 different subcutaneous PDAC PDX tumours treated as indicated when the tumours reached 100mm^3^ in NOD-SCIDg mice. Dosages of each agent were: ulixertinib 100 mg/kg BID orally 5 days/week, afatinib 12.5 mg/kg orally daily, copanlisib 10 mg/kg by tail vein injection two times/week, gemcitabine 75 mg/kg by intraperitoneal injection once per week. N=4-6 tumours per treatment. Data was presented as mean±SEM. *P*-values were calculated using Mixed-effect analysis with Tukey's multiple comparisons test. WU-0002: gemcitabine vs ulixertinib + gemcitabine (*p*=0.424), gemcitabine vs ulixertinib + afatinib + gemcitabine (*p*=0.018), gemcitabine vs ulixertinib + copanlisib + gemcitabine (*p*=0.0058), wu-0003: gemcitabine vs ulixertinib + gemcitabine (*p*=0.895), gemcitabine vs ulixertinib + afatinib + gemcitabine (*p*=0.003), gemcitabine vs ulixertinib + copanlisib + gemcitabine (*p*=0.003), wu-0004: gemcitabine vs ulixertinib + gemcitabine (*p*=0.8714), gemcitabine vs ulixertinib + afatinib + gemcitabine (*p*=0.0047), gemcitabine vs ulixertinib + copanlisib + gemcitabine (*p*=0.0022), wu-0006: gemcitabine vs ulixertinib + gemcitabine (*p*=0.9971), gemcitabine vs ulixertinib + afatinib + gemcitabine (*p*=0.0488), gemcitabine vs ulixertinib + copanlisib + gemcitabine (*p*=0.001), wu-0007: gemcitabine vs ulixertinib + gemcitabine (*p*<0.0001), gemcitabine vs ulixertinib + afatinib + gemcitabine (*p*<0.0001), gemcitabine vs ulixertinib + copanlisib + gemcitabine (*p*<0.0001), wu-0009: gemcitabine vs ulixertinib + gemcitabine (*p*=0.4044), gemcitabine vs ulixertinib + afatinib + gemcitabine (*p*=0.0075), gemcitabine vs ulixertinib + copanlisib + gemcitabine (*p*=0.0459), wu-0011: gemcitabine vs ulixertinib + gemcitabine (*p*=0.042), gemcitabine vs ulixertinib + afatinib + gemcitabine (*p*<0.0001), gemcitabine vs ulixertinib + copanlisib + gemcitabine (*p*=0.0001), wu-0016: gemcitabine vs ulixertinib + gemcitabine (*p*=0.8803), gemcitabine vs ulixertinib + afatinib + gemcitabine (*p*=0.0035), gemcitabine vs ulixertinib + copanlisib + gemcitabine (*p*=0.0029), wu-0018; gemcitabine vs ulixertinib + gemcitabine (*p*=0.052), gemcitabine vs ulixertinib + afatinib + gemcitabine (*p*=0.002), gemcitabine vs ulixertinib + copanlisib + gemcitabine (*p*=0.002), wu-0022: gemcitabine vs ulixertinib + gemcitabine (*p*=0.0825), gemcitabine vs ulixertinib + afatinib + gemcitabine (*p*=0.035), gemcitabine vs ulixertinib + copanlisib + gemcitabine (*p*=0.0002), wu-0023: gemcitabine vs ulixertinib + gemcitabine (*p*=0.042), gemcitabine vs ulixertinib + afatinib + gemcitabine (*p*<0.0001), gemcitabine vs ulixertinib + copanlisib + gemcitabine (*p*<0.0001), wu-0044: gemcitabine vs ulixertinib + gemcitabine (*p*=0.0018), gemcitabine vs ulixertinib + afatinib + gemcitabine (*p*<0.0001), gemcitabine vs ulixertinib + copanlisib + gemcitabine (*p*<0.0001), wu-0060: gemcitabine vs ulixertinib + gemcitabine (*p*=0.7842), gemcitabine vs ulixertinib + afatinib + gemcitabine (*p*=0.3500), gemcitabine vs ulixertinib + copanlisib + gemcitabine (*p*=0.2945), wu-0066: gemcitabine vs ulixertinib + gemcitabine (*p*=0.9974), gemcitabine vs ulixertinib + afatinib + gemcitabine (*p*=0.0001), gemcitabine vs ulixertinib + copanlisib + gemcitabine (*p*=0.0001), wu-0068: gemcitabine vs ulixertinib + gemcitabine (*p*=0.3323), gemcitabine vs ulixertinib + afatinib + gemcitabine (*p*=0.9767), gemcitabine vs ulixertinib + copanlisib + gemcitabine (*p*=0.0048), wu-0105: gemcitabine vs ulixertinib + gemcitabine (*p*=0.3023), gemcitabine vs ulixertinib + afatinib + gemcitabine (*p*=0.033), gemcitabine vs ulixertinib + copanlisib + gemcitabine (*p*=0.048). **b**, serial body weights of NOD-SCIDg mice bearing different PDAC xenografts monitored from the initiation to the end of the indicated treatment. Source data are provided in source data file.

**Supplementary Figure 5**

**
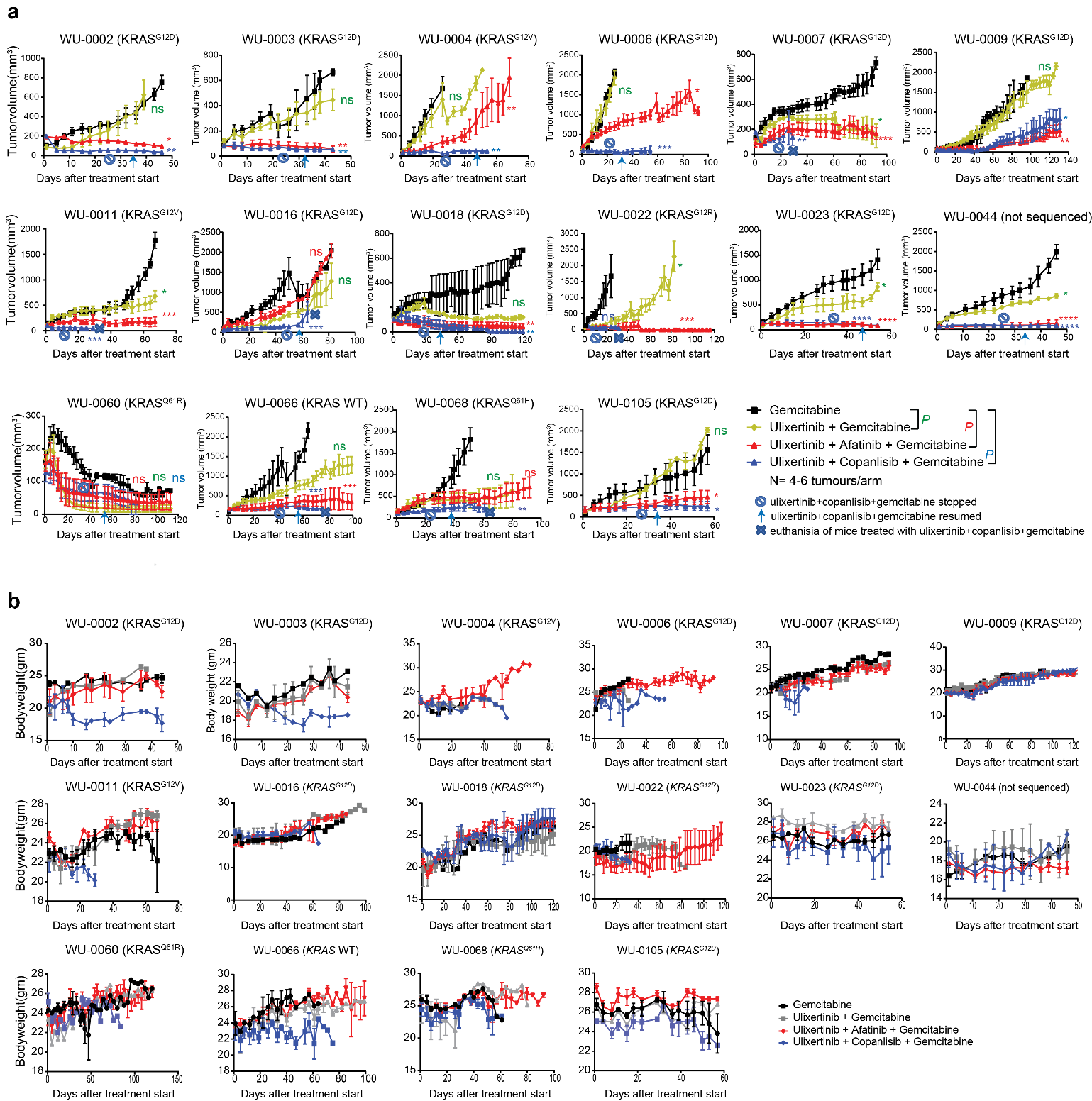
**

**Supplementary Figure 6. MAPK inhibitors plus trastuzumab deruxtecan (DS-8201a) lead to deep and durable treatment response.**

**a,** Serial body weights of NOD-SCIDγ mice bearing different PDAC xenografts from monitored from the initiation to the end of the indicated treatment. **b,** Serum chemistry of mice treated as indicated for at least 2 weeks. Blood draw was performed from tail vein and collected sample processed into serum for storage in -80°C prior to analysis. Blood samples from two independent experiments were tested and presented. N=3-8 per arm, and *p*-values were calculated using two-way ANOVA followed by Tukey’s multiple comparison test (ns, not significant). In the box plots, the center line represents the median whereas the lower and higher lines correspond to the first and third quartiles, respectively. The whisker extends from the hinge to at most interquartile range (IQR). **c,** Representative H&E images of different organs of mice treated as indicated till humane endpoints. Scale bars=400 μM. At least 10 mice/group were examined by a board-certified pathologist (MBR) and no gross abnormality was seen. Source data are provided in Source Data file.

**Supplementary Figure 6**

**
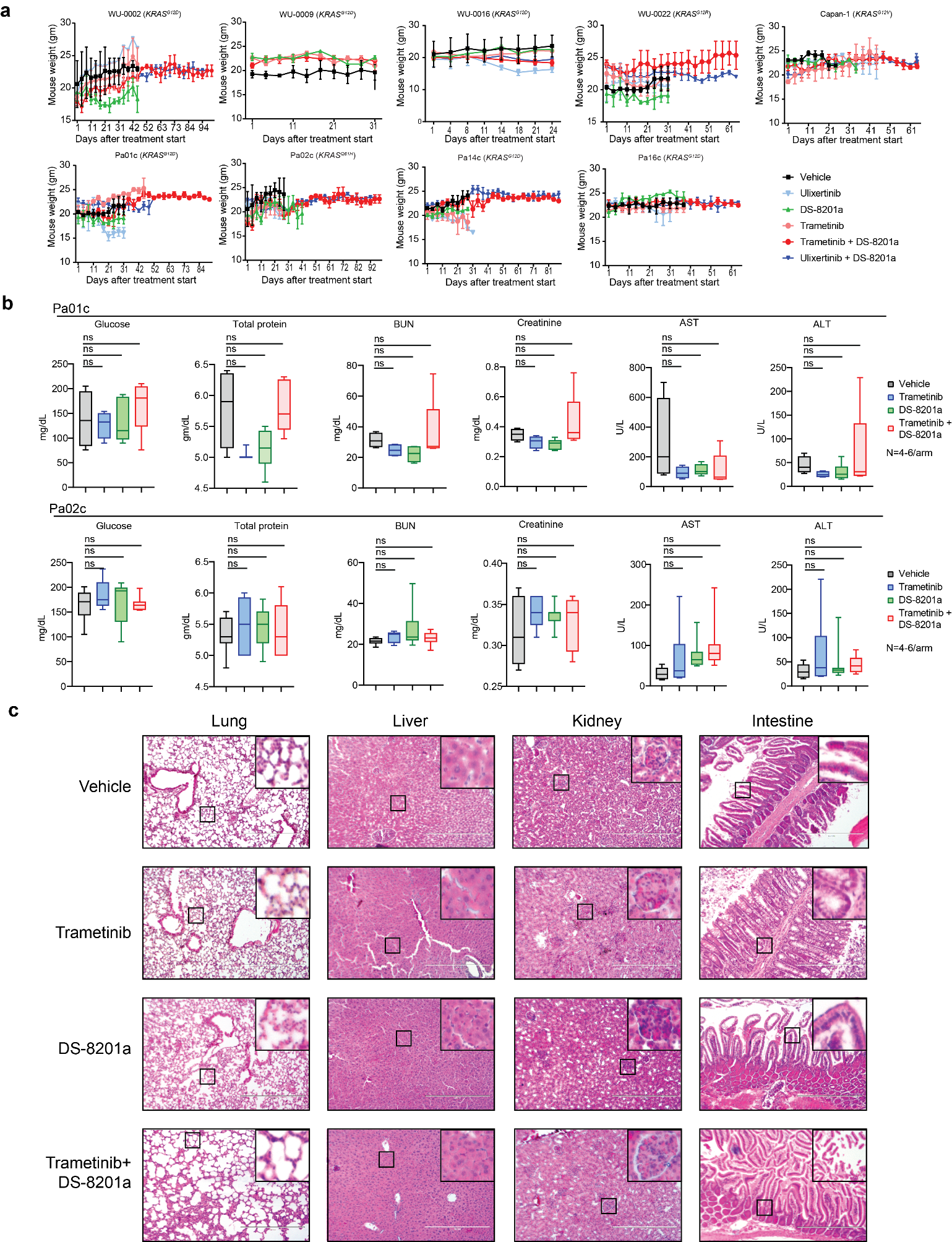
**

**Supplementary Figure 7. Different MEK inhibitors also upregulate HER2 expression.**

**a,** Western blots showing changes of phosphorylated HER2 in two different PDAC cells treated with the indicated MEK inhibitors for 16 h. (**a**) was conducted two times, and one set of data was presented. **b,** Representative FACS plots and quantification showing changes in surface (without cell permeabilization) and total (with cell permeabilization) HER2 abundance following 16 h treatment with different MEK inhibitors in two different *KRAS^G12D^*-mutant cell lines. Data represents one of three independent experiments each done in triplicates. Data was presented as mean±SEM. *P*-values were calculated using one-way ANOVA with Dunnet’s multiple comparisons test. For Pa01c surface HER2 (1µM or 2µM Selumetinib vs Vehicle (*p<*0.0001), 100nM or 500nM Mirdametinib vs Vehicle (*p<*0.0001)), and Total HER2 (1µM Selumetinib vs Vehicle (*p=*0.2571), 2µM Selumetinib vs Vehicle (*p*=0.0074); 100nM Mirdametinib vs Vehicle (*p=*0.0008), 500nM Mirdametinib vs Vehicle (*p<*0.0001). For HPAC Surface HER2 (1µM or 2µM Selumetinib vs Vehicle (*p<*0.0001), 100nM or 500nM Mirdametinib vs Vehicle (*p<*0.0001)), and Total HER2 (1µM Selumetinib vs Vehicle (*p=*0.2571), 2µM Selumetinib vs Vehicle (*p*=0.0074); 100nM Mirdametinib vs Vehicle (*p=*0.0008), 500nM Mirdametinib vs Vehicle (*p<*0.0001). Source data are provided as a Source Data file

**Supplementary Figure 7**


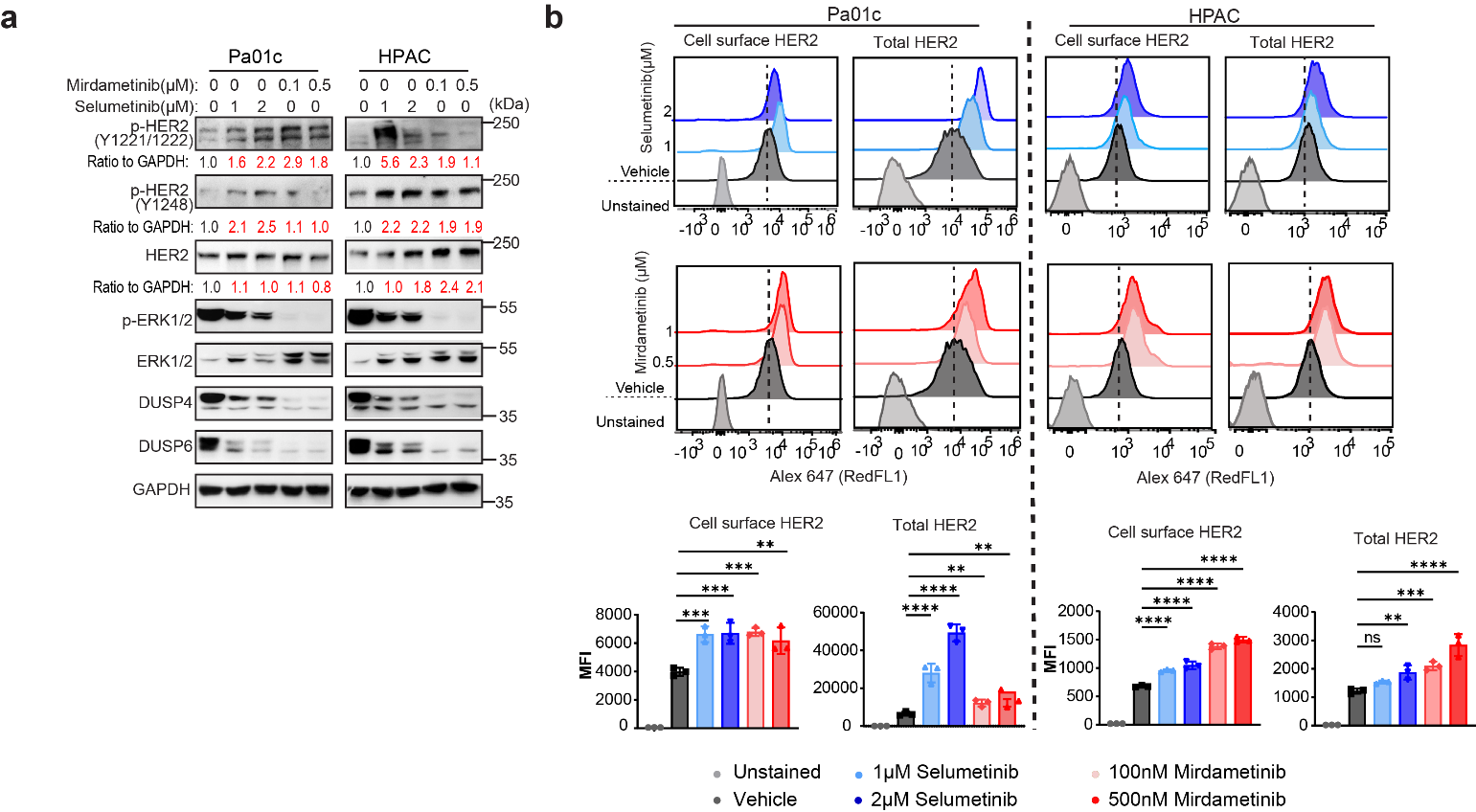


**Supplementary Figure 8. KRAS inhibitors plus trastuzumab deruxtecan (DS-8201a) showed promising preclinical efficacy.**

**a,** Representative FACS plots and quantification showing changes in surface (without cell permeabilization) and total (with cell permeabilization) HER2 abundance following 16 h treatment as indicated in two different *KRAS^G12C^*-mutant colon cancer cell lines. (AMG-510 0.5μM, DS-8201a 0.1μg/ml). n= three independent experiment. Data was presented as mean±SEM. P-values were calculated using one-way ANOVA with Dunnet’s multiple comparisons test. For SW837 Surface HER2 (AMG-510 vs Vehicle (*p<*0.0001), DS-8201a vs Vehicle (*p*<0.0001) & DS-8201a+AMG-510 vs Vehicle (*p*<0.0001) and Total HER2 (AMG-510 vs Vehicle (*p*=0.08), DS-8201a vs Vehicle (*p*<0.0001), DS-8201a+AMG-510 vs AMG-510 (*p*<0.0001) and DS-8201a+AMG-510 vs DS-8201a (*p*= 0.2075). For SW1463 Surface HER2 (AMG-510 vs Vehicle (*p*=0.0097), DS-8201a vs Vehicle (*p*<0.0001), DS-8201a+AMG-510 vs AMG-510 (*p*<0.0001) & DS-8201a+AMG-510 vs DS-8201a (*p*=0.2226), and Total HER2 (AMG-510 vs Vehicle (*p*=0.1233), DS-8201a vs Vehicle (*p*<0.0001), DS-8201a+AMG-510 vs AMG-510 (*p*<0.0001) and DS-8201a+AMG-510 vs DS-8201a (*p*=0.8858). **b,** Dose-dependent curves for the inhibitory effect of MRTX1133 in the indicated scramble or *ERBB*-silenced Pa01c or HPAC cells lines following 5 days of treatment. Three independent experiments were performed, yielding similar results and IC_50_ were provided from one set. Source data are provided as a Source Data file.

**Supplementary Figure 8**

**
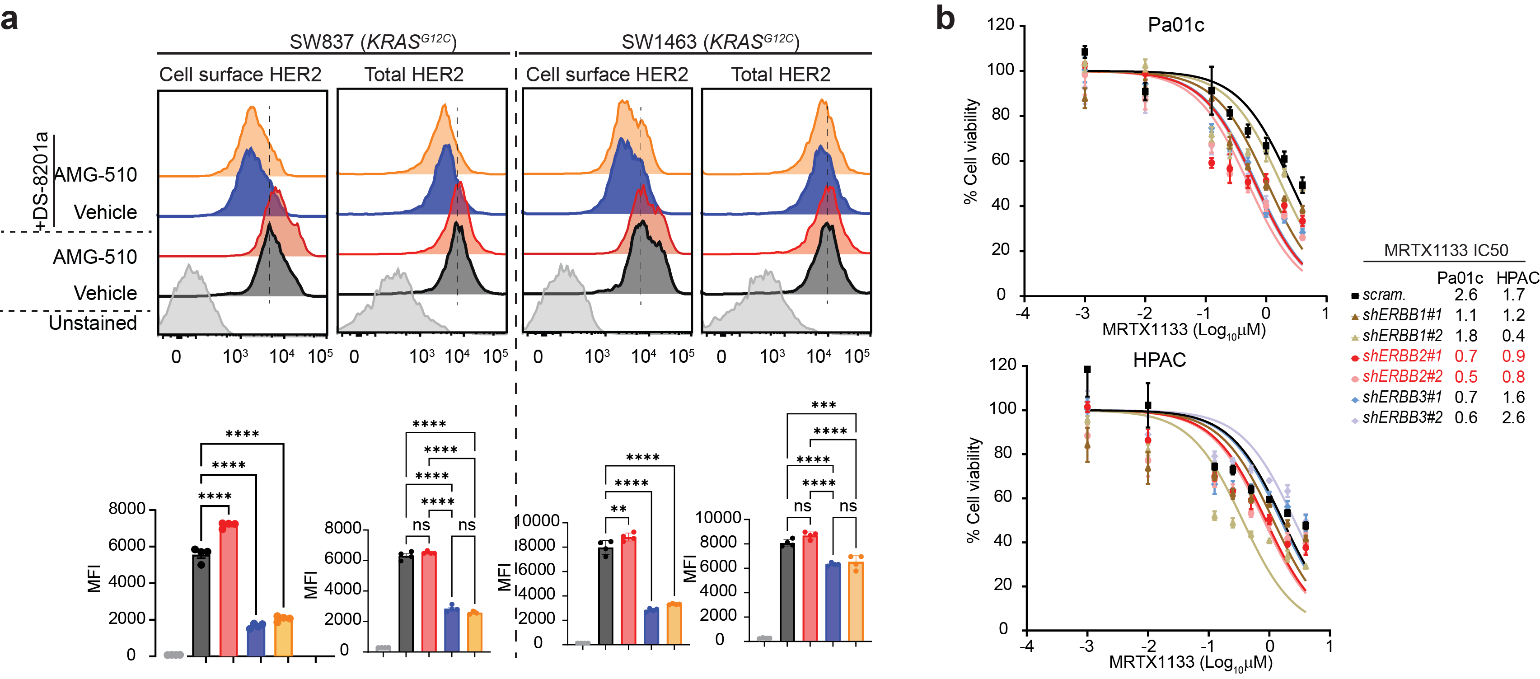
**

**Supplementary Figure 9**. **Gating strategy used in FACS data analysis.**

**a**, Representative FACS plots showing the gating strategy used in each experiment to characterize apoptotic cells in response to different drug treatments using Annexin-V and PI co-staining. PI/ or Annexin V single stain on fixed dead cells were used to identify apoptotic positive cell population. Annexin V positive apoptotic cells can be seen in the bottom right quadrant and both annexin V and PI positive dead cells are seen in the top right quadrant. **b**, Representative gating strategy for single color stain (HER2) samples and unstained controls.

**Supplementary Figure 9**


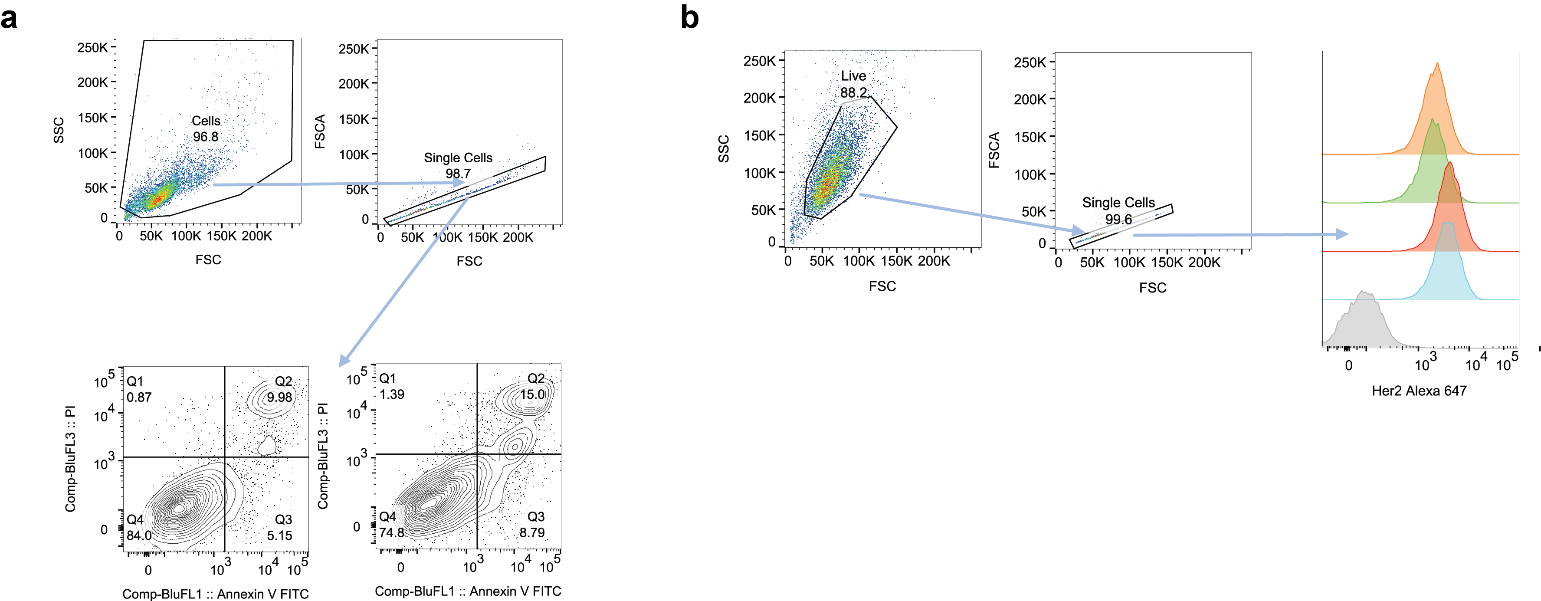


**Supplementary Table 1: Primary antibodies used**

| **Supplementary Table 1-Primary antibodies** | | | | | |
| --- | --- | --- | --- | --- | --- |
| **Name** | **Host** | **Clone#** | | **Company** | **Dilution** |
| Phospho-AKT(S473) | Rabbit | 4060S | | Cell signaling | Western blot-1:1000 |
| AKT | Rabbit | 4691S | | Cell signaling | Western blot-1:1000 |
| Cleaved caspase-3 | Rabbit | 9661S | | Cell signaling | Western blot-1:1000 |
| PARP | Rabbit | 9542S | | Cell signaling | Western blot-1:1000 |
| Phospho-EGFR (Y1068) | Rabbit | 3777S | | Cell signaling | Western blot-1:1000 |
| EGFR | Rabbit | 4267S | | Cell signaling | Western blot-1:1000 |
| Phospho-HER2 (Y1248) | Rabbit | 2247S | | Cell signaling | Western blot-1:1000 |
| Phospho-HER2 (Y1221/1222) | Rabbit | 2243S | | Cell signaling | Western blot-1:1000 |
| Phospho-HER2 (Y1196) | Rabbit | 6942S | | Cell signaling | Western blot-1:1000 |
| Phospho-HER2 (Y877) | Rabbit | 2241S | | Cell signaling | Western blot-1:1000 |
| Phospho-HER2 (T686) | Rabbit | 88583S | | Cell signaling | Western blot-1:1000 |
| Alexa fluor647 anti-human CD340 (erbB2/HER-2) | Mouse | 324D2 | | BioLegend | FACS (2ul per test) |
| HER2 | Rabbit | 4290S | | Cell signaling | Western blot-1:1000;IHC-1:300 |
| Phospho-HER3 (Y1197) | Rabbit | 4561S | | Cell signaling | Western blot-1:1000 |
| HER3 | Rabbit | 12708S | | Cell signaling | Western blot-1:1000 |
| DUSP1/MKP-1 (D-3) | mouse | sc-271684 | | Santa cruz | Western blot-1:500 |
| DUSP3/VHR | mouse | NBP2-59460 | | Novus biologicals | Western blot-1:500 |
| DUSP4/MKP-2 | Rabbit | 5149S | | Cell signaling | Western blot-1:1000;IHC-1:200 |
| DUSP5 (H-9) | mouse | sc-39380 | | Santa cruz | Western blot-1:500 |
| DUSP6/MKP-3 (F-12) | mouse | sc-377070 | | Santa cruz | Western blot-1:500;IHC-1:200 |
| DUSP7/PYST2 (D-8) | mouse | sc-271684 | | Santa cruz | Western blot-1:500 |
| DUSP8 (B-9) | mouse | sc-271250 | | Santa cruz | Western blot-1:500 |
| DUSP9/MKP-4(D-3) | mouse | sc-137010 | | Santa cruz | Western blot-1:500 |
| DUSP10/MKP-5(G-10) | mouse | sc-374276 | | Santa cruz | Western blot-1:500 |
| GAPDH (O411) | Rabbit | sc-47724 | | Santa cruz | Western blot-1:1000 |
| GFP | Rabbit | NB600-308 | | Novus | Western blot-1:1000 |
| Flag | Rabbit | 14793S | | Cell signaling | Western blot-1:1000 |
| HA | Rabbit | 3724S | | Cell signaling | Western blot-1:1000 |
| Phospho-ERK1/2(Thr202/Tyr204) | Rabbit | 4370S | | Cell signaling | Western blot-1:1000 |
| ERK1/2 | Rabbit | 4695S | | Cell signaling | Western blot-1:1000;IHC-1:300 |
| Phospho-MEK1/2(S217/221) | Rabbit | 9154S | | Cell signaling | Western blot-1:1000 |
| MEK1/2 | Rabbit | 9122S | | Cell signaling | Western blot-1:1000 |
| KRAS | Mouse | F264 | | Santa cruz | Western blot-1:500 |
| Phospho-RSK(S380) | Rabbit | 9344S | | Cell signaling | Western blot-1:1000 |
| RSK | Rabbit | 5528S | | Cell signaling | Western blot-1:1000 |
| TRIM21 | Rabbit | 92043S | | Cell signaling | Western blot-1:1000 |
| Ubiquitin | Rabbit | 43124S | | Cell signaling | Western blot-1:1000 |
| Ubiquitin (K48) | Rabbit | 8081S | Cell signaling | | Western blot-1:1000 |

**Supplementary Table 2: Oligo sequence used**

| **Name** | **Gene** | **Species** | **Clone ID** | **Target sequence** |
| --- | --- | --- | --- | --- |
| shERBB1#1 | ERBB1 | Human | TRCN0000039635 | CCCGTCGCTATCAAGGAATTA |
| shERBB1#2 | ERBB1 | Human | TRCN0000121069 | CGCAAAGTGTGTAACGGAATA |
| shERBB2#1 | ERBB2 | Human | TRCN0000219708 | AGCCTTCGACAACCTCTATTA |
| shERBB2#2 | ERBB2 | Human | TRCN0000196927 | GAGATCACAGGTTACCTATAC |
| shERBB3#1 | ERBB3 | Human | TRCN0000000622 | CGACTAGACATCAAGCATAAT |
| shERBB3#2 | ERBB3 | Human | TRCN0000194972 | CTTCGTCATGTTGAACTATAA |
| shDUSP4#1 | DUSP4 | Human | TRCN0000368367 | CGCAGTTCGTCTTCAGCTTTC |
| shDUSP4#2 | DUSP4 | Human | TRCN0000244861 | GGCAATAAGGACTCCGAATAC |
| shDUSP6#1 | DUSP6 | Human | TRCN0000378156 | GCATCTGGAACTGACTATATA |
| shDUSP6#2 | DUSP6 | Human | TRCN0000355536 | CTGATACTCCATTTGATTATT |
| shTRIM21#1 | Trim21 | Human | TRCN0000234745 | TGGAAGTGGAAATTGCAATAA |
| shTRIM21#2 | Trim21 | Human | TRCN0000234748 | GAAGAGAGATTTGATAGTTAT |

**Supplementary Table 3: Plasmids used.**

| **Name of insert** | **Source** | **Catalogue number** |
| --- | --- | --- |
| HA-DUSP6 | Addgene | 27975 |
| HA-DUSP6 (C293S) | Addgene | 27977 |
| HA-DUSP7 | Addgene | 27976 |
| pLVXpuro-TRIM21-GFP | Addgene | 116941 |
| pLVXpuro-TRIM21(C54Y)-GFP | Addgene | 116942 |
| FLAG-HER1-pcDNA3.1 | Amplified by PCR and cloned into empty vector |  |
| FLAG-HER2-pcDNA3.1 | Amplified by PCR and cloned into empty vector |  |
| FLAG-HER2-pcDNA3.1 | Amplified by PCR and cloned into empty vector |  |
| FLAG-DUSP4-pWZL-blast | Amplified by PCR and cloned into empty vector |  |
| FLAG-DUSP6-pWZL-blast | Amplified by PCR and cloned into empty vector |  |
| HER2C-term676-end-pET-29b(+) | Synthesized byTwist Bioscience |  |
| HER2C-term676-end-TEYAAmutant-pET-29b(+) | Synthesized byTwist Bioscience |  |
| ERK2TEYAAmutant-pET-29b(+) | Synthesized byTwist Bioscience |  |
| ERK2WT-pET-29b(+) | Synthesized byTwist Bioscience |  |

**Supplementary Table 4: Drugs and serial concentrations used.**

|  | | |  |  |  |  |
| --- | --- | --- | --- | --- | --- | --- |
|  |  |  |  |  |  |  |
| **Drug** | **Serial concentrations used** | | | | | |
| Ulixertinib(μM) | 20 | 10 | 2 | 1 | 0.5 | 0.25 |
| Trametinib(μM) | 5 | 2.5 | 0.5 | 0.25 | 0.125 | 0.0625 |
| Afatinib(μM) | 5 | 2.5 | 0.5 | 0.25 | 0.125 | 0.0625 |
| MMPi(μM) | 20 | 10 | 2 | 1 | 0.5 | 0.25 |
| MUCi(μM) | 10 | 5 | 1 | 0.5 | 0.25 | 0.125 |
| Ruxolitinib(μM) | 20 | 10 | 2 | 1 | 0.5 | 0.25 |
